# Afterload-related cardiac performance identifies cardiac impairment and associates with outcome in patients with septic shock: a retrospective cohort study

**DOI:** 10.1186/s40560-021-00549-5

**Published:** 2021-04-13

**Authors:** Wei-yan Chen, Zhen-hui Zhang, Li-li Tao, Qi Xu, Xing Wei, Min-sheng Chen

**Affiliations:** 1 Department of Cardiology, Heart Center, Zhujiang Hospital, Southern Medical University, Guangzhou, China; 2 Intensive Care Unit, the Second Affiliated Hospital of Guangzhou Medical University, Guangzhou, China; 3 Guangdong Provincial Biomedical Engineering Technology Research Center for Cardiovascular Disease, Guangzhou, China; 4 Sino-Japanese Cooperation Platform for Translational Research in Heart Failure, Guangzhou, China; 5 Laboratory of Heart Center, Zhujiang Hospital, Southern Medical University, Guangzhou, China

**Keywords:** Afterload-related cardiac performance, Septic cardiomyopathy, Mortality, Cardiac index, Cardiac power index

## Abstract

**Background:**

Septic patients with cardiac impairment are with high mortality. Afterload-related cardiac performance (ACP), as a new tool for diagnosing septic cardiomyopathy (SCM), still needs to be evaluated for its impact on the prognosis for patients with septic shock.

**Methods:**

In this retrospective study, 100 patients with septic shock undertaken PiCCO monitoring were included. The ability of ACP, cardiac index (CI), and cardiac power index (CPI) to discriminate between survivors and non-survivors was tested by comparing the area under the receiver operating characteristic curve (AUROC) analysis. Cox proportional hazards regression analyses were performed to assess the associations of ACP with day-28 mortality. Curve estimation was used to describe the relationship between the hazard ratio (HR) of death and ACP.

**Results:**

ACP had a strong linear correlation with CI and CPI (*P* < 0.001). ACP demonstrated significantly greater discrimination for day-28 mortality than CI before adjusted [AUROC 0.723 (95% CI 0.625 to 0.822) vs. 0.580 (95% CI 0.468 to 0.692), *P* = 0.007] and CPI after adjusted [AUROC 0.693 (95% CI 0.590 to 0.797) vs*.* 0.448 (0.332 to 0.565), *P* < 0.001]. Compared with ACP > 68.78%, HR for ACP ≤ 68.78% was 3.55 (1.93 to 6.54) (*P* < 0.001). When adjusted with age, APACHE-II score, Vasoactive Inotropic Score, Lactate, CRRT, day-1 volume, fibrinogen and total bilirubin as possible confounders, and decrease ACP are still associated with increasing day-28 mortality (*P* < 0.05). An exponential relationship was observed between ACP12h and HR of day-28 death.

**Conclusions:**

Our results suggested thatACP could improve mortality predictions when compared to CI and CPI. Decreased ACP was still an independent risk factor for increased day-28 mortality.

**Supplementary Information:**

The online version contains supplementary material available at 10.1186/s40560-021-00549-5.

## Background

Sepsis is caused by a dysregulated host response to infection, which leads to life-threatening organ dysfunction [1]. The heart is one of the most frequently affected organs. It has been known for years that severe impairment of cardiac function is not only one of the leading causes of septic shock, but also contributes to mortality in the intensive care unit (ICU) [2]. However, septic cardiomyopathy (SCM) was difficult to define because of its limited means of diagnosis and inconsistent criteria in the last few decades. As a result, the prevalence reported varies from studies [3, 4]. It was usually recognized only when obvious cardiac dysfunction was present in the clinical condition. The mechanism of SCM and its influence on prognosis are also not well understood.

Many attempts have been made to early recognize and quantify the severity of SCM, for example, left ventricular ejection fraction (LVEF), cardiac index (CI), myocardial performance index (MPI), and others. Nevertheless, “the impaired cardiac function” in septic patients is often masked by the severe reduction of afterload, which leads to a compensatory increase of cardiac output (CO) and LVEF. Several studies reported that when SCM was defined by echocardiography, LVEF was not associated with in-hospital and 30-day mortality in patients with sepsis or septic shock [5–7]. In a meta-analysis, there were no significant differences in LVEF, right ventricular ejection fractions, and right ventricular dimensions between the survivor and non-survivor groups [8]. Su et al. demonstrated that only low CI combined with high stroke volume variation increased mortality [9]. Accordingly, LVEF and CI are not ideal indicators for SCM.

In recent years, the strain measured by speckle tracking technique (STT) is considered less susceptible to changes in pre- or afterload [10–12]. In a multi-center prospective cohort study, Chang et al demonstrated that global longitudinal strain was an independent prognostic indicator of ICU mortality [13]. However, STT still carries a disadvantage of being a discontinuous measurement. The process of SCM is still unclear. When to take the STT is indeed an important problem that complicates the investigators. The afterload-related cardiac performance (ACP), first introduced by Werdan et al. in 2011 [14], is a quantitative measure of SCM. It is a ratio of measured to predicted cardiac output, which represents the cardiac ability to increase its output when systemic vascular resistance (SVR) decreases in order to maintain a constant mean arterial pressure (MAP). These measures are obtained from an indicator-dilution or pulse contour analytic cardiac output monitoring device. It proposes an option for a more relevant continuous monitoring of cardiac performance than is currently available. It is reported that ACP correlated well with 30-day mortality when calculated on admission in patients with community-acquired sepsis [15]. ACP may be a potential effective means for SCM diagnosis, but still need more studies to reveal the relationship between ACP and SCM.

The primary aims of this study were to assess the effect of a decrease in ACP within the first 24 h of septic shock in discriminating against day-7, day-14, and day-28 mortality.

## Methods

### Setting

This was a retrospective cohort study, approved by The Second Affiliated Hospital of Guangzhou Medical University Clinical Research and Application Institutional Review Board in Guangzhou, China. The Second Affiliated Hospital of Guangzhou Medical University is a tertiary hospital with a 32-bed multidisciplinary ICU. The ICU has an electronic patient record system where most of the data is recorded at the time of generation.

### Patients and study design

Adult patients (aged ≥ 18 years) undertaken pulse indicator continuous cardiac output technology (PiCCO®, Pulsion, Munich, Germany) in the first 24-h time period of septic shock during his/her stay in ICU between June 2016 and June 2019 were screened for study inclusion. Septic shock was defined as a subset of sepsis and clinically identified by a vasopressor requirement to maintain a MAP of 65 mm Hg or greater and serum lactate level greater than 2 mmol/L in the absence of hypovolemia (sepsis-3) [1]. Patients were excluded if they met one of the following criteria: [1] repeat ICU admissions from the same hospital episode [2]; previous history of significant underlying cardiac conditions, such as ischemic cardiac disease, congenital heart disease, severe valvular heart disease, and cardiomyopathy [3]; active diagnoses directly relating to myocardial dysfunction, such as acute myocardial infarction, myocarditis, myocardial effusion, unstable arrhythmia, and post-cardiopulmonary resuscitation status [4]; effective PiCCO monitor less than 24 h.

In our ICU, parameters estimated by PiCCO were measured and recorded every 6 h. MAP was monitored continuously and recorded hourly.

### Data collection

We extracted the following data: demographics, chronic co-morbidities [coronary heart disease (CHD), chronic kidney disease (CKD), diabetes, hypertension], Acute Physiology and Chronic Health Evaluation (APACHE) II score, fluid challenge (total amount) and output, and fluid load (volume = total amount - output), and the use of vasoactive agents and Vasoactive Inotropic Score (VIS) was calculated [VIS = 100 × norepinephrine (μg/kg/min) + 100 × epinephrine (μg/kg/min) + 10 × milrinone (ng/kg/min) + 1 × dopamine (μg/kg/min) + 1 × dobutamine (μg/kg/min)] [16]. The laboratory measurements, such as blood routine examination, hepatic function, renal function, coagulation function, serum procalcitonin (PCT), and lactate levels on the first day of septic shock, were collected. LVEF was estimated by transthoracic echocardiography within 48 h of septic shock. The focus of infection attributed to septic shock was collected. We also extracted data on the type of organ support, for example, the application of mechanical ventilation and continuous renal replacement therapy (CRRT). CO, CI, cardiac power index (CPI), global end-diastolic volume index (GEDI), systemic vascular resistance index (SVRI), extravascular lung water index (ELWI), central venous pressure (CVP), and MAP were collected at 0h, 6h, 12h, 18h, and 24h after PiCCO monitoring. Survival status on day-7, day-14, and day-28 after septic shock, ICU, and hospital discharge were collected. ICU length to stay (LOS), hospital LOS, and LOS of 28 days after septic shock were also collected.

Missing data for all screening variables was less than 20% (Table S1). Assuming that data was missing at random, missing data was imputed via the method of expectation and maximization.

### Afterload-related cardiac performance (ACP)

ACP is described as CO_measured_/CO_predicted as normal_ × 100. It was calculated using the formula previously described by Werdan et al.: ACP (%) = 100 × CO/[560.68 × ((MAP - CVP) × 80/CO)^-0.645^]. ACP was classified as normal (> 80%), slight impairment (60% ~ 80%), moderate impairment (40% ~ 60%), and severe impairment (< 40%), respectively.

### Outcomes

The primary study outcome was to explore the prognostic accuracy of ACP, CI, and CPI for day-28 mortality among critically ill patients with septic shock. The secondary study outcomes included day-7 and day-14 mortality.

### Statistical analysis

Continuous variables with normal distribution were summarized as mean and standard deviation (SD), otherwise median and inter-quartile range (IQR, 25th percentile to 75th percentile). Normal distribution was tested by Kolmogorov-Smirnov test. Categorical variables were described as frequencies or percentages. Group comparisons were conducted using Fisher’s exact tests for equal proportions, *t* tests for normally distributed data, and Mann-Whitney’s *U* tests otherwise. The ability of ACP, CI, and CPI to discriminate between survivors and non-survivors was tested by comparing the area under the receiver operating characteristic curve (AUROC) analysis (unadjusted analysis) and adjusted with a baseline risk model (adjusted analysis). Specific AUROC (95% CI) values were generated. The cut-off value was defined by the maximum of the sum of sensitivity and specificity.

To further assess the associations of ACP with day-28 mortality, multivariable logistic regression and Cox proportional hazards regression analyses were performed. A Scatter diagram was drawn, and curve estimation was used to describe the relationship between hazards ratio (HR) of death and ACP. The assumption of linearity for the continuous variable was assessed by the Martingale Residual test. Multicollinearity was assessed using the Pearson correlation coefficient statistics and by checking the Variance Inflation Factor on a multiple regression model with the same dependent and independent variables. The proportional hypothesis was assessed using the Schoenfeld Individual Test. The likelihood ratio test was used to test the overall significance of the model. The fit of the model was assessed by the Concordance Index.

A two-tailed *p* value < 0.05 was set as statistically significant. All analyses were performed using SPSS 22.0 and R. software version 4.0.2.

## Results

### Prognostic predictive value of ACP at different points of time

**Fig. 1:**
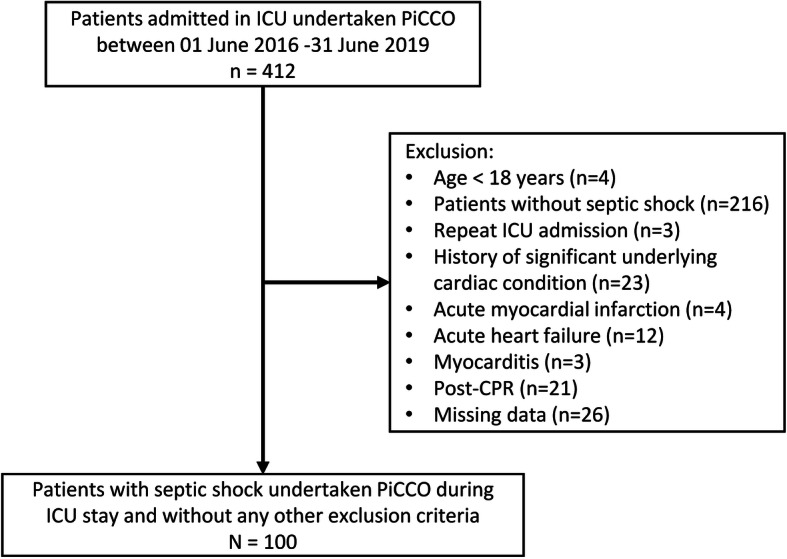
Patient flow chart. ICU intensive care unit, PiCCO pulse indicator continuous cardiac output technology, CPR cardiopulmonary resuscitation

Between June 2016 and June 2019, 412 patients were undertaken PiCCO in the ICU; 216 patients did not have septic shock and 96 patients met one of the exclusion criteria; at last, 100 patients were included (Fig. 1).

In order to find out ACP measured at which time point had the best prognostic predictive value, crude AUROCs at each time point within the first 24 h of septic shock were calculated (Table 1). It is found that ACP assessed at 12 h (ACP12h) had the highest AUROC not only in day-7 and day-14 mortality prediction but also day-28 mortality prediction. The cut-off value for day-28 mortality prediction was 68.78%.

**Table 1:** Discriminative abilities of ACP assessed at different time points (*n*=100)

	ACP0h	ACP6h	ACP12h	ACP18h	ACP24h
**Day-7 mortality**					
Crude AUROC (95% CI)	0.593 (0.474–0.712)	0.713 (0.606–0.820)	0.816 (0.728–0.903)	0.709 (0.583–0.834)	0.760 (0.647–0.873)
*P* value	0.128	0.001	<0.001	0.001	<0.001
**Day-14 mortality**					
Crude AUROC (95% CI)	0.616 (0.506–0.726)	0.703 (0.601–0.805)	0.792 (0.706–0.879)	0.682 (0.571–0.793)	0.699 (0.586–0.812)
*P* value	0.046	<0.001	<0.001	0.002	0.001
**Day-28 mortality**					
Crude AUROC (95% CI)	0.576 (0.462–0.689)	0.643 (0.625–0.822)	0.723 (0.625–0.822)	0.620 (0.510–0.730)	0.675 (0.563–0.786)
*P* value	0.197	0.014	<0.001	0.041	0.004

### Demographic data and main measurements of patients with septic shock

Among this cohort, 90 (90.0%) patients had low ACP within the first 24 h of septic shock, of which 54 (54.0%) patients were slightly impaired, 32 (32.0%) patients were moderately impaired, and 4 (4.0%) were severely impaired. The majority of patients with moderately to severely myocardial impaired (80.6%) died 28 days after septic shock. While patients with normal ACP only had low mortality (10.0%) before 14 days after septic shock (Figure S1). However, only 15 (12.61%) patients had abnormal LVEF (<50%) and 45 (45.0%) patients had low CI (< 3.0 L/min/m^2^).

Patients with ACP12h less than 68.78% were characterized by significantly older, a higher APACHE-II score and VIS, higher level of CVP, total bilirubin and serum lactate, lower MAP within the first 24 h of septic shock, and greater need for CRRT and fluid resuscitation (day-1 total amount) compared to patients with ACP12h more than 68.78%. Fifteen patients (35.7%) with ACP12h more than 68.78% died in the ICU. On the other hand, only fifteen patients (25.9%) with ACP12h less than 68.78% survived in the ICU. There was no difference in GEDI, SVRI, and ELWI between the two groups (Table 2).

**Table 2:** Demographic data and main measurements among septic shock patients

	All (*N*=100)	ACP12h≤68.78% (***n***=58)	ACP12h>68.78% (***n***=42)	***P*** value
**Demographics**				
Age, mean (SD), year	67.7 (15.4)	70.3 (15.2)	63.9 (15.0)	0.040
Male, No. (%)	62 (62.0)	33 (56.9)	29 (69.0)	0.217
BMI, mean (SD), kg/m^2^	21.7 (4.2)	21.6 (4.3)	21.7 (4.2)	0.877
**Severity of illness on the beginning of septic shock, mean (SD) or median (IQR)**				
APACHE-II score	23.8 (8.9)	25.4 (9.1)	21.7 (8.3)	0.037
Vasoactive inotropic score	103.5 (20.8–174.5)	137.2 (73.4–220.9)	27.7 (0.7–102.5)	< 0.001
**Vital signs, mean (SD) or median (IQR)**				
MAP, mmHg	80.1 (13.2)	72.9 (9.9)	90.0 (10.6)	<0.001
Respiratory rate, times/min	26.0 (20.0–34.8)	25.0 (20.0–32.0)	29.5 (20.8–37.3)	0.135
Heart rate, beats/min	117.2 (26.8)	117.4 (27.6)	117.0 (26.0)	0.948
Body temperature, °C	37.0 (36.2–38.0)	37.0 (36.3–38.0)	37.0 (36.0–38.0)	0.916
**Focus of infection, NO. (%)**				
Bloodstream infection	18 (18.0)	9 (15.5)	9 (21.4)	0.448
Pulmonary infection	37 (37.0)	19 (32.8)	18 (42.9)	0.302
Abdominal infection	23 (23.0)	13 (22.4)	10 (23.8)	0.870
Urinary infection	3 (3.0)	3 (5.2)	0 (0.0)	0.135
Infection of biliary tract	7 (7.0)	5 (8.6)	2 (4.8)	0.455
Skin soft-tissue infection	1 (1.0)	1 (1.7)	0 (0.0)	0.392
Other	11 (11.0)	8 (13.8)	3 (7.1)	0.294
**Medical history, No. (%)**				
Chronic heart disease	17 (17.0)	12 (20.7)	5 (11.9)	0.248
Chronic kidney disease	10 (10.0)	6 (10.3)	4 (9.5)	0.893
Diabetics	12 (12.0)	7 (12.1)	5 (11.9)	0.980
Hypertension	12 (12.0)	6 (10.3)	6 (14.3)	0.549
**Combined therapy**				
Mechanical ventilation, No. (%)	95 (95.0)	55 (94.8)	40 (95.2)	0.926
CRRT, No. (%)	59 (59.0)	40 (69.0)	19 (45.2)	0.017
Day-1 total amount, mean (SD), ml^a^	3935.2 (1500.9)	4365.6 (1568.3)	3340.8 (1181.4)	0.001
Day-1 output, mean (SD), ml^b^	2447.8 (1386.5)	2385.6 (1379.9)	2533.9 (1407.7)	0.600
Day-1 volume, mean (SD), ml^c^	1487.3 (1831.0)	1980.0 (1932.3)	806.9 (1444.5)	0.001
**Outcomes**				
Day-7 mortality, No. (%)	34 (34.0)	30 (51.7)	4 (9.5)	<0.001
Day-14 mortality, No. (%)	47 (47.0)	39 (67.2)	8 (19.0)	<0.001
Day-28 mortality, No. (%)	57 (57.0)	43 (74.1)	14 (33.3)	<0.001
ICU mortality, No. (%)	58 (58.0)	43 (74.1)	15 (35.7)	< 0.001
ICU LOS, median (IQR), d	9.0 (5.0–17.8)	8.0 (3.0–13.3)	12.5 (6.8–20.8)	0.018
Hospital mortality, No. (%)	61 (61.0)	44 (75.9)	17 (40.5)	< 0.001
Hospital LOS, median (IQR), d	22.0 (11.0–32.8)	15.5 (6.8–28.0)	23.5 (15.5–40.3)	0.006
**PICCO data, mean (SD) or median (IQR)**				
CVP, cmH_2_O	14.7 (5.9)	16.0 (6.1)	12.9 (5.2)	0.009
GEDI, ml/m^2^	707.0 (605.5–842.3)	698.0 (599.3–861)	712.0 (616.8–817.5)	0.992
SVRI, dyn.s.cm^-5^.m^2^	2063.7 (792.6)	2119.7 (921.3)	1986.5 (571.1)	0.376
ELWI, ml/kg	9.0 (6.6–14.5)	9.2 (7.0–15.0)	8.2 (6.0–13.6)	0.206
CI, L/min/m^2^	3.09 (0.98)	2.77 (0.98)	3.55 (0.79)	<0.001
CPI, W/m^2^	0.55 (0.20)	0.44 (0.16)	0.70 (0.16)	<0.001
**LVEF, median (IQR), %**				
LVEF	60.0 (55.0–64.8)	58.7 (54.0–63.3)	62.0 (59.4–65.3)	0.006
**Laboratory test, mean (SD) or median (IQR)**				
White blood cell, ×10^9^/L	10.8 (6.1–19.1)	12.8 (6.2–21.2)	8.7 (5.8–15.6)	0.180
Neutrophils lymphocytes ratio	21.8 (26.4)	22.7 (27.6)	20.6 (24.9)	0.702
Hemoglobin, g/L	94.6 (24.4)	92.1 (23.8)	98.1 (25.0)	0.229
Platelet, ×10^9^/L	99.0 (41.0–191.5)	72.5 (33.8–186.8)	129.5 (54.8–208.3)	0.051
Total bilirubin, μmol/L	23.5 (13.0–45.5)	35.0 (13.8–55.7)	16.7 (11.0–29.3)	0.005
Albumin, g/L	25.3 (4.8)	24.6 (4.9)	26.4 (4.6)	0.061
Serum creatinine, μmol/L	156.9 (92.4–229.5)	161.2 (95.9–234.7)	125.6 (86.6–222.1)	0.311
Urea, mmol/L	12.0 (8.0–17.9)	11.8 (7.4–19.0)	12.2 (8.1–16.0)	0.810
Calcium ion, mmol/L	1.98 (1.87–2.17)	2.00 (1.89–2.17)	1.97 (1.87–2.18)	0.722
D-dimer, mg/L	5.3 (3.1–10.0)	5.1 (3.2–15.8)	6.2 (2.8–8.4)	0.603
Fibrinogen, g/L	3.6 (1.8)	3.3 (1.7)	4.1 (1.8)	0.023
Procalcitonin, ng/ml	24.4 (3.0–56.7)	31.6 (5.3–58.4)	19.0 (1.0–56.4)	0.336
Lactate, mmol/L	4.8 (2.0–8.9)	5.6 (2.3–12.1)	3.8 (1.9–5.6)	0.006

^a^Day-1 total amount, the total amount of fluid in the first day

^b^Day-1 output, the total output in the first day

^c^Day-1 volume, the fluid load in the first day =Day-1 total amount–Day-1 output

### Correlation between ACP and traditional parameters

**Fig. 2:**
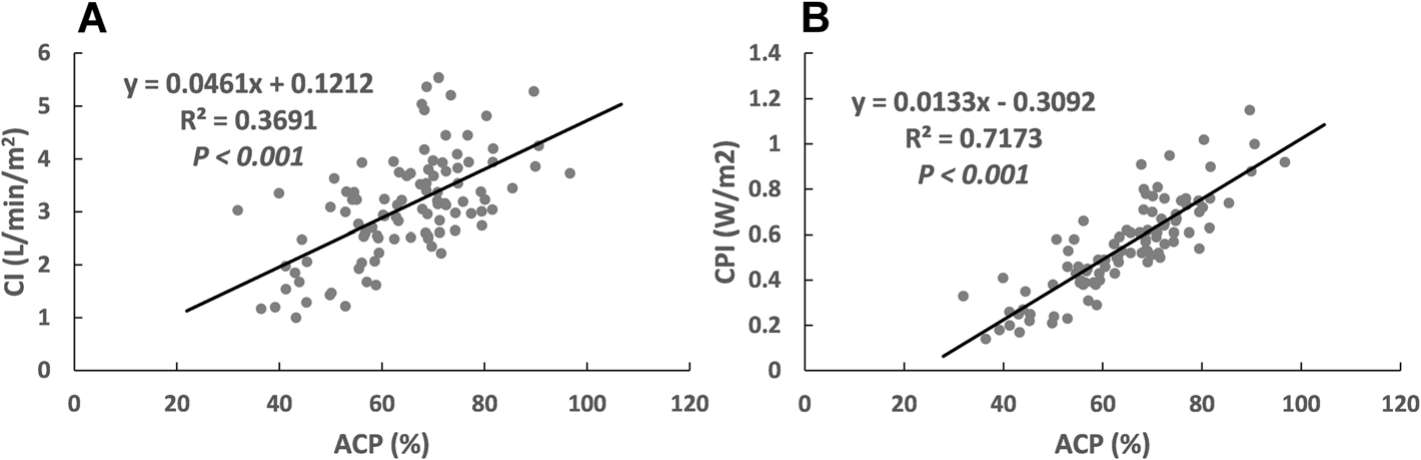
Correlation of ACP with traditional parameters. Calculations are based on all values collected at 12h after septic shock. CI cardiac index, ACP afterload-related cardiac performance, CPI cardiac power index

To investigate if ACP could be used to quantify the severity of cardiac impairment, we looked at the correlation between ACP and traditional parameters reflecting cardiac function. It is found that ACP showed strong correlations to CI, which was an important indicator for the diagnosis of cardiogenic shock (Fig. 2a). Furthermore, ACP showed significant correlations to CPI, an indicator of ventricular arterial coupling (Fig. 2b).

### Prognostic predictive value of ACP, CI, and CPI

**Fig. 3:**
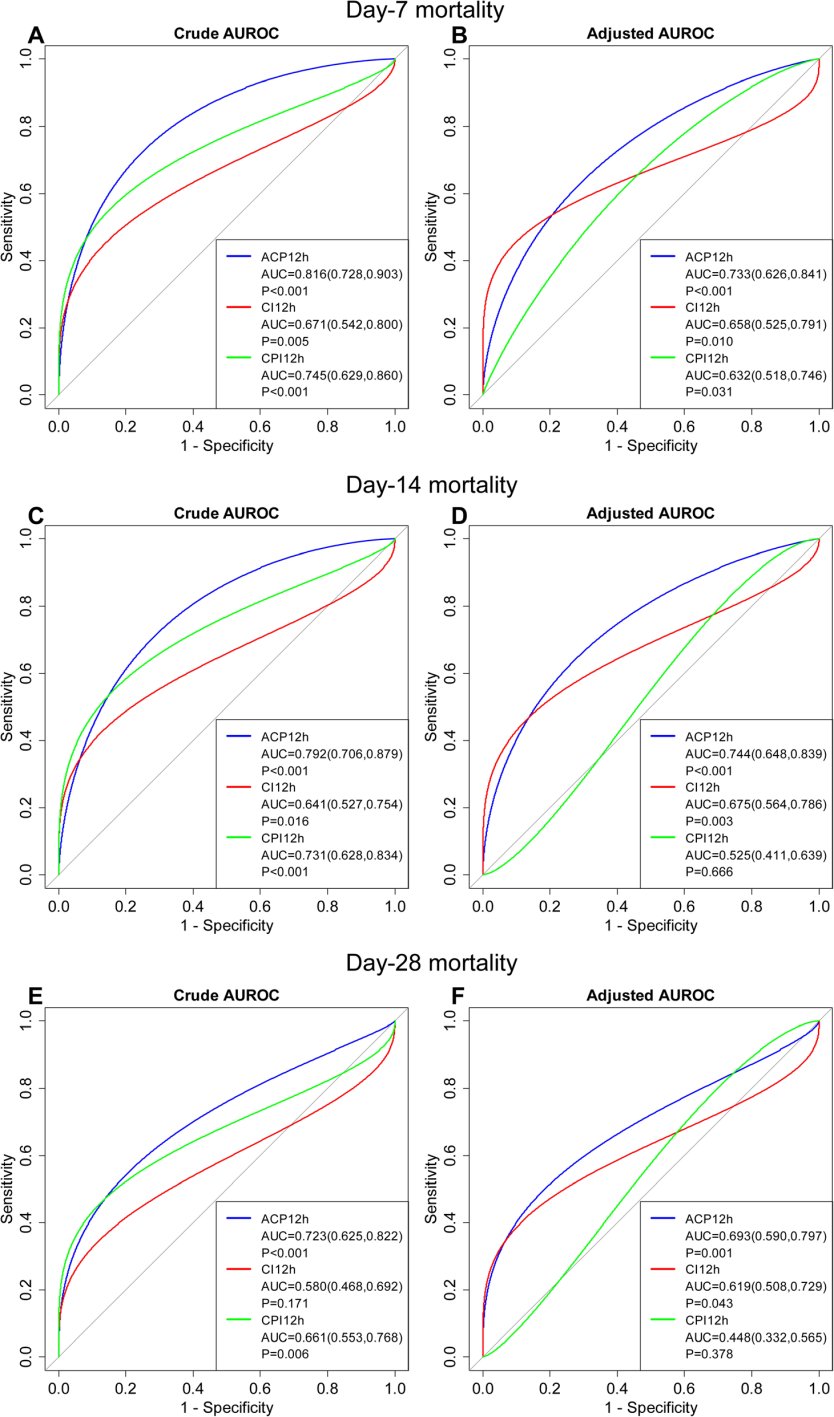
Area under the receiver operating characteristic curves (AUROCs) for day-7, day-14, and day-28 mortality for ACP, CI, and CPI

Discrimination of day-28 mortality after septic shock was significantly higher using ACP12h than CI12h with all incremental differences being statistically significant (*P* < 0.01) (Fig. 3e). With a cut-off value of 68.78% or below, ACP12h predicted non-survival at day-28 with a sensitivity of 75.4%, a specificity of 65.1%, a positive predictive value (PPV) of 74.1%, a negative predictive value (NPV) of 66.7%, and accuracy of 71.0% (Table S2). While adjusted with age (*P* = 0.03), APACHE-II score (*P* < 0.001), VIS (*P* < 0.001), CRRT (*P* < 0.001), day-1 volume (*P* = 0.021), and lactate (*P* = 0.01) as possible confounders (Table S5), ACP12h outperformed CPI12h for discrimination of day-28 mortality of septic shock with incremental differences being statistically significant (*P* < 0.001) (Fig. 3f).

The superior discriminatory performance of ACP12h was maintained when considering the secondary outcomes of day-7 and day-14 mortality when considered in isolation or adjusted with the baseline prediction (Fig. 3a–d, Table S3 and S4).

### Cox proportional hazards regression analyses of day-28 mortality according to ACP12h

**Fig. 4:**
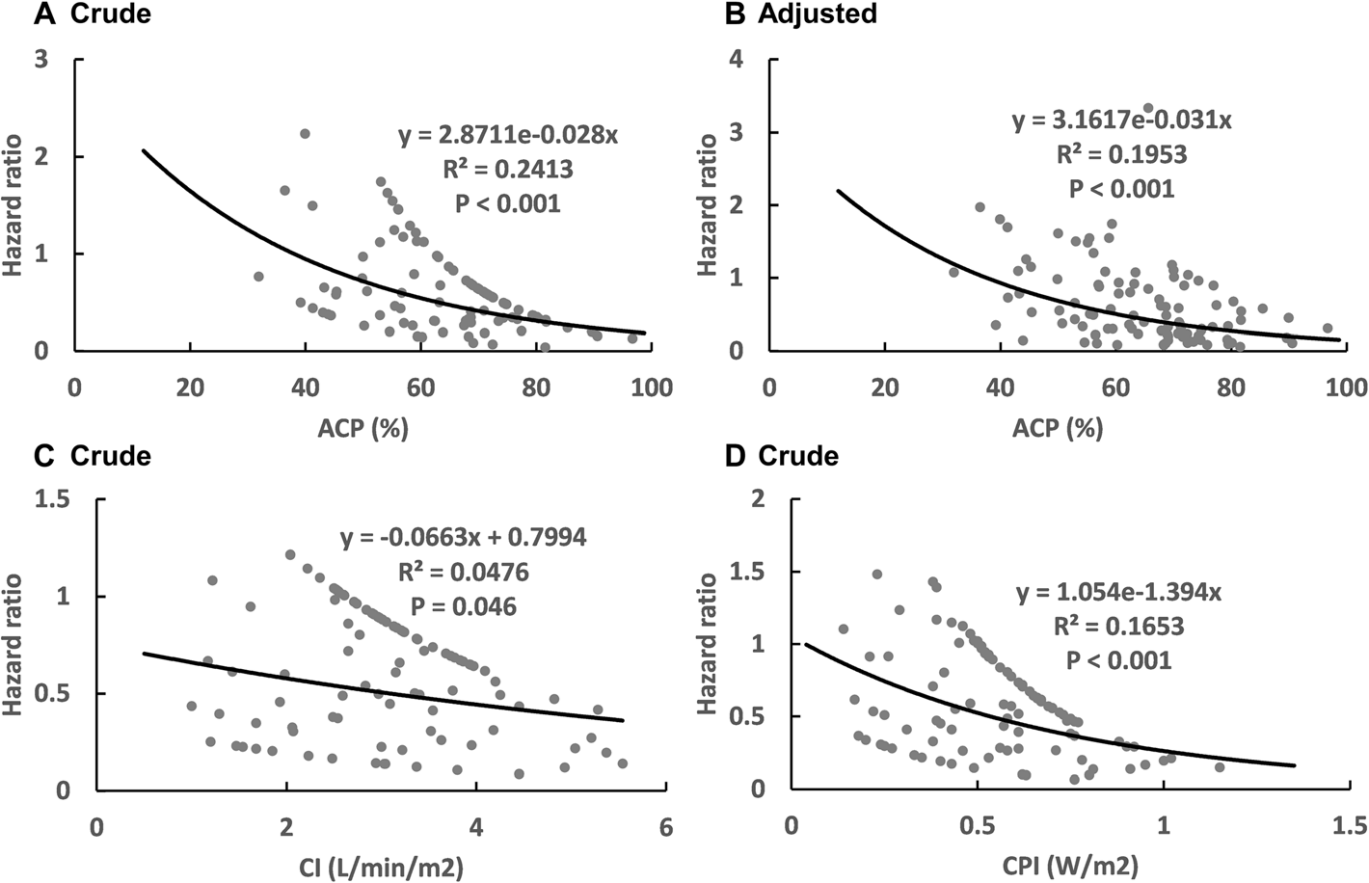
Relationship between parameters of cardiac function and hazard ratio of day-28 mortality in patients with septic shock. **a** Crude model of relationship between ACP12h and HR. **b** Adjusted model of relationship between ACP12h and HR, after adjustment for baseline risk of day-28 mortality (age, APACHE-II score, VIS, lactate, CRRT, day-1 volume, fibrinogen and total bilirubin). **c**. Crude model of relationship between CI12h and HR. **d**. Crude model of  relationship between CPI12h and HR

As a continuous variable, reduced HR of day-28 death was significantly associated with increased levels of ACP12h, CI12h, and CPI12h (Table 3). An exponential relationship was observed between ACP12h and HR of day-28 death (Fig. 4a). An exponential relationship was also observed between CI12h and HR of day-28 death, CPI12h, and HR of day-28 death (Fig. 4c, d). After adjusting for risk factors, reduced HR of day-28 death was still significantly associated with increased levels of ACP12h, while CI12h and CPI12h were not. An exponential relationship was also observed between ACP12h and HR of day-28 death (Fig. 4b).

**Table 3:** Cox proportional hazards regression analyses of 28-day mortality according to ACP12h, CI12h and CPI12h (*n* = 100)

Variable	**ACP12h (%)**		**CI12h (L/min/m**^**2**^**)**		**CPI12h (W/m**^**2**^**)**	
	> 68.78	≤ 68.78	>2.5	≤ 2.5	> 0.46	≤ 0.46
**Continuous variable, HR (95% CI)**						
Crude model	0.94 (0.92–0.96) *P* < 0.001		0.72 (0.52–0.99) *P* = 0.041		0.05 (0.01–0.26) *P* < 0.001	
Adjusted model	0.97 (0.94–0.99) *P* = 0.012		0.98 (0.71–1.34) *P* = 0.882		0.30 (0.06–1.57) *P* = 0.154	
**Classifications of variable by cut-off value, HR (95% CI)**						
Crude model	1.00	3.55 (1.93–6.54) *P* < 0.001	1.00	3.19 (1.81–5.61) *P* < 0.001	1.00	5.04 (2.93–8.69) *P* < 0.001
Adjusted model	1.00	2.29 (1.14–4.60) *P* = 0.021	1.00	2.02 (1.04–3.90) *P* = 0.038	1.00	3.53 (1.79–7.0) *P* < 0.001

Adjusted model: age, APACHE-II score, VIS, Lactate, CRRT, day-1 volume, fibrinogen, and total bilirubin

We further analyzed ACP12h, CI12h, and CPI12h as categorical variables in a Cox model. Using patients with ACP > 68.78% as the reference group, HRs were 3.553 (1.931 to 6.539) for ACP ≤ 68.78% group. Using patients with CI > 2.5 L/min/m^2^ as the reference group, HRs were 3.188 (1.812 to 5.608) for CI ≤ 2.5 L/min/m^2^ group. Using patients with CPI > 0.46 W/m^2^ as the reference group, HRs were 5.044 (2.927 to 8.693) for CPI ≤ 0.46 W/m^2^ group. Similar findings were also observed for day-28 mortality regardless of HR adjusted for risk factors (Table 3 and Figure S2, S3, S4).

## Discussion

In this retrospective study, mortality at 28 days was 57%, much higher than previous reports 30–42% [17, 18]. This may have something to do with the fact that patients selected for PiCCO monitoring were severe. We can find that the APACHE-II score and VIS were high in this population, which demonstrated that most patients presented with severe hemodynamic instability.

Our study found that ACP showed a strong correlation with CI and CPI. CI has been proposed as a helpful tool to detect impaired cardiac function in heart failure. CPI obtained by CO×MAP×0.0022 was proved to be a good hemodynamic parameter to identify cardiac reserve [19]. Low CPI resulted in an increased mortality rate [20]. Therefore, ACP may be a useful parameter reflecting the severity of cardiac impairment.

Unlike other types of cardiomyopathy, patients with SCM have extensive vascular hyporesponsiveness to catecholamine in addition to impaired cardiac function [21]. Studies found that patients with septic shock often had a normal or elevated CI due to the decrease in SVR [9, 22]. Therefore, the CI obviously cannot truly and comprehensively reflect the cardiac function impairment of patients in septic shock. Naturally, the predictive value of CI in the prognosis of SCM will also be greatly reduced. By correcting the afterload, ACP makes up for the deficiency of CI to some extent and may be more sensitive and accurate in reflecting the cardiac function impairment in patients with sepsis. In Wilhelm et al.’s study, it is demonstrated that only ACP was the hemodynamic parameter predicting mortality and significantly influenced by the severity of sepsis, whereas CI was not [15]. In our study, discrimination of day-28 mortality after the septic shock was significantly higher using ACP12h than CI12h. These suggest ACP is superior to CI for predicting outcomes in sepsis. The role of CPI, which is calculated using MAP just as ACP, has not been evaluated in detail. It is reported that CPI had no predictive value for mortality at the early stage of sepsis [15]. While our study found that, with a cut-off value of 0.46W/m^2^ or below, CPI12h predicted non-survival with a high specificity as 95.3%, but a low sensitivity (only 50.9%) at the late stage of sepsis (septic shock). It demonstrated that a low CPI implies worse outcomes in patients with septic shock, but it may not be a sensitive tool for SCM diagnosis. In comparison with CPI, a decrease in ACP demonstrated superior prognostic accuracy for day-7, day-14, and day-28 mortality. This may be related to the fact that ACP is not only corrected by MAP, but also by CVP. As far as we know, MAP is only one component of the afterload. It generally decreases significantly until the late stage of sepsis, so MAP is not sensitive to reflect the change of afterload at the early stage of sepsis. Therefore, the predictive ability of CPI for outcome at the early stage of sepsis may not be as strong as it is at the late stage of sepsis.

The APACHE-II score, an important indicator of disease severity, has been proved to be a good predictor for outcome in sepsis [23]. While ACP is an indicator of the severity of cardiac impairment, which is not included in the APACHE-II score. Our study found that the superior discriminatory performance of ACP12h for day-28 mortality was maintained when adjusted with APACHE-II score and other confounders. The combination of APACHE-II score and ACP will increase the accuracy of the prediction.

Furthermore, unlike Wilhelm et al.’s study, which used 80% as the cut-off point, our study found that patients with mild impairment ACP did not have a significantly increased risk of day-28 mortality compared with patients with normal ACP (Figure S5). Our study demonstrated that ACP at or lower than 68.78% was still an independent risk factor for day-28 mortality with HR 3.55 (95%CI 1.93–6.54) (*P* <0.001). The 28-day mortality was 74.1% in the ACP ≤ 68.78% group, whereas 33.3% in the ACP > 68.78% group. All of the deaths in 28 days occurred during the ICU period (Table 2). Especially most patients in the ACP ≤ 68.78% group died within 1 to 2 weeks. Most studies have suggested that SCM usually recovers within 2 weeks after infection control and the mortality decreased significantly after recovery of cardiac function. Therefore, as an early stage parameter, ACP12h not only had superior prognostic accuracy for day-7 and day-14, but also for day-28 mortality, which is a late stage parameter.

As a continuous variable, an exponential relationship was observed between ACP12h and HR of day-28 death. Although an exponential relationship was also observed between CI12h and HR of day-28 death, and CPI12h and HR of day-28 death, the curves fitted poorly and results were hard to explain. From Fig. 4, we can find that HR was still less than 1 when CI or CPI was low. Therefore, it is not a suitable model to describe the relationship between CI12h and HR of day-28 death, and CPI12h and HR of day-28 death.

In general, ACP may be a useful tool for quantifying cardiac impairment in sepsis and predicting outcome. However, ACP has the disadvantage of not accounting for preload. Therefore, preload independence must be assessed correctly before ACP measurement is standardized. In our study, all patients included were screened for the sufficiency of early fluid resuscitation through the record to minimize the impact of preload. Our results demonstrated that there was no difference in GEDI and SVRI between ACP ≤ 68.78% group and ACP > 68.78% group. Day-1 total amount reached 4365.6 ± 1568.3 ml in ACP ≤ 68.78% group and 3340.8 ± 1181.4 ml in ACP > 68.78% group. In the prediction model and cox model, we also adjusted for the confounders including day-1 volume, inotropes, and vasoactive agents. In addition, ACP assessed at 12 h had better discriminative ability than other time points might be related to the fact that ACP at this time point reflects the intrinsic cardiac function due to volume having been restored after adequate fluid resuscitation.

This study had a number of strengths. Compared to the study published by Wilhelm et al., the population in our study involved only septic shock patients, which was more severe, with a higher incidence of SCM and a higher mortality. Our study brings back to the forefront a forgotten hemodynamic index and demonstrated that it may be a useful indicator of SCM and a good predictor for mortality through establishing relationship between ACP and traditional parameters and comparing it to them. Moreover, to the best of our knowledge, this is the first study to compare different time points at assessing cardiac function by ACP in patients with septic shock.

### Limitations

Several limitations must be considered in our study. First, this was a small retrospective cohort study, limited factors could be studied, and the preload independence before ACP measurements could not be assured. Second, our finding was based on data obtained from patients undertaken PiCCO monitoring, which might lead to selection bias and impacted by unmeasured confounders. The third limitation was that the predictive model needs validated by validation cohort. Fourth, ACP is also a non-automated measurement, and it is not clear whether its calculation formula needs to be corrected among different ethnic groups.

### Clinical perspective

There are few studies on ACP at present. ACP as a potential diagnostic method of SCM has not yet been widely accepted. More large-scale studies are needed to provide evidence for this, particularly those that identify the diagnostic accuracy of ACP for cardiac function by comparison with the golden standard. In recent years, some retrospective studies have found that extracorporeal membrane oxygenation (ECMO) can significantly improve the survival rate of patients with refractory shock caused by sepsis-induce myocardial dysfunction, and the efficacy was better than that of patients with only vascular hyporesponsiveness [24–26]. ACP, corrected for afterload, may be a useful tool for distinguishing SCM from vascular hyporesponsiveness and identifying patients with sepsis induced refractory shock who are suitable for ECMO treatment. ECMO treatment may be a potential treatment for patients with refractory shock caused by a remarkable decrease in ACP.

## Conclusions

In conclusion, our results suggest that the ACP at or lower than 68.78% was an independent risk factor for mortality of 28 days. The assessment of ACP at 12 h after septic shock in ICU significantly improves day-7, day-14, and day-28 mortality predictions when compared to CI and CPI. With the decline in ACP, the HR of day-28 death in patients with septic shock increased exponentially.

## Supplementary Information

**Additional file 1.** Supplement file.

## Data Availability

The datasets used and analyzed during the current study are available from the corresponding author on reasonable request.

## Abbreviations

ACP: Afterload-related cardiac performance
SCM: Septic cardiomyopathy
CI: Cardiac index
LVEF: Left ventricular ejection fraction
AUROC: Area under the receiver operating characteristic curve
HR: Hazard ratio
ICU: Intensive care unit
CO: Cardiac output
STT: Speckle tracking technique
SVR: Systemic vascular resistance
PiCCO: Pulse indicator continuous cardiac output technology
CPI: Cardiac power index
MAP: Mean arterial pressure
CHD: Coronary heart disease
CKD: Chronic kidney disease
APACHE-II: Acute Physiology and Chronic Health Evaluation
VIS: Vasoactive inotropic score
PCT: Procalcitonin
WBC: White blood cell
CRRT: Continuous renal replacement therapy
CVP: Central venous pressure
LOS: Length of stay
SD: Standard deviation
PPV: Positive predictive value
NPV: Negative predictive value
