# Supplementary material for: Afterload-related cardiac performance identifies cardiac impairment and associates with outcome in patients with septic shock: a retrospective cohort study
Source: J Intensive Care. 2021 Apr 13;9:33. doi: 10.1186/s40560-021-00549-5 (PMC8042871; doi:10.1186/s40560-021-00549-5)

Table S1. All screening variables contained less than 20% missing values

Variables	Missing number (%)
Age	0 (0)
Gender	0 (0)
BMI	0 (0)
APACHEII score	0 (0)
VIS	0 (0)
Respiratory rate	0 (0)
Heart rate	0 (0)
MAP	0 (0)
Body temperature	0 (0)
Focus of infection	0 (0)
Medical history	0 (0)
Mechanical ventilation	0 (0)
CRRT	0 (0)
Day-1 volume	0 (0)
ICU length of stay	0 (0)
Hospital length of stay	0 (0)
PiCCO data	0 (0)
LVEF	20 (20)
White blood cell	0 (0)
Neutrophils lymphocytes ratio	0 (0)
Hemoglobin	0 (0)
Platelet	0 (0)
Total bilirubin	11 (11)
Albumin	11 (11)
Serum creatinine	1 (1)
Urea	1 (1)
Calcium ion	1 (1)
D-dimer	8 (8)
Fibrinogen	6 (6)
Procalcitonin	16 (16)
Lactate	18 (18)

Abbreviation: BMI, body mass index; APACHEII score, Acute Physiology and Chronic Health Evaluation II score; VIS, Vasoactive Inotropic Score; MAP, mean arterial pressure; CRRT, continuous renal replacement therapy; LVEF, left ventricular ejection fraction.

Table S2 Crude and adjusted AUROCs for discrimination characteristics of ACP, CI, and CPI among patients with septic shock (n = 100).

	ACP12h	CI12h	CPI12h	Between, group difference		
				ACP12h vs. CI12h	ACP12h vs. CPI12h	CI12h vs. CPI12h
**Day-7 mortality**						
Crude AUROC (95% CI)	0.816 (0.728-0.903)	0.671 (0.542-0.800)	0.745 (0.629-0.860)	0.145 (0.035-0.255)	0.071 (0-0.173)	0.074 (0-0.196)
Cut-off (Spe, Se)	62.60 (0.758, 0.735)	2.50 (0.924, 0.500)	0.46 (0.864, 0.647)	--	--	--
P-value	<0.001	0.005	<0.001	0.008	0.064	0.006
Adjusted AUROC (95% CI)	0.733 (0.626-0.841)	0.658 (0.525-0.791)	0.632 (0.518-0.746)	0.075 (0-0.196)	0.101 (0.010-0.212)	0.026 (0-0.150)
P-value	<0.001	0.010	0.031	0.216	0.016	0.619
**Day-14 mortality**						
Crude AUROC (95% CI)	0.792 (0.706-0.879)	0.641 (0.526-0.755)	0.731 (0.628-0.834)	0.151 (0.050-0.252)	0.061 (0-0.156)	0.090 (0-0.199)
Cut-off (Spe, Se)	68.78 (0.642, 0.830)	2.50 (0.943, 0.404)	0.46 (0.906, 0.553)	--	--	--
P-value	<0.001	0.016	<0.001	0.004	0.090	0.001
Adjusted AUROC (95% CI)	0.744 (0.648-0.839)	0.675 (0.564-0.786)	0.525 (0.411-0.639)	0.069 (0-0.173)	0.219 (0.114-0.324)	0.150 (0.037-0.263)
P-value	<0.001	0.003	0.666	0.210	<0.001	0.005
**Day-28 mortality**						
Crude AUROC (95% CI)	0.723 (0.625-0.822)	0.580 (0.468-0.692)	0.661 (0.553-0.768)	0.143 (0.039-0.247)	0.062 (0-0.165)	0.081 (0-0.191)
Cut-off (Spe, Se)	68.78 (0.651, 0.754)	2.50 (0.930, 0.333)	0.46 (0.953, 0.509)	--	--	--
P-value	<0.001	0.171	0.006	0.007	0.081	0.005
Adjusted AUROC (95% CI)	0.693 (0.590-0.797)	0.619 (0.508-0.729)	0.448 (0.332-0.565)	0.074 (0-0.181)	0.245 (0.135-0.355)	0.171 (0.057-0.285)
P-value	0.001	0.043	0.378	0.150	<0.001	<0.05

Abbreviation: Spe, specificity; Se, sensitivity; 95% CI: 95% confidence interval.

[a] Baseline risk factors for the model to predict day-7 mortality including age, APACHEII score, VIS, CRRT, day-1 volume, platelet and lactate.

[b] Baseline risk factors for the model to predict day-14 mortality including age, APACHEII score, VIS, CRRT, day-1 volume, platelet, total bilirubin, fibrinogen and lactate.

[c] Baseline risk factors for the model to predict day-28 mortality including age, APACHEII score, VIS, CRRT, day-1 volume and lactate.

Table S3 Covariates for the baseline risk prediction model for day-7 mortality

Variable	Survivors (n=66)	Non-survivors (n=34)	Odds Ratio (95% CI)	*P*-value
**Age Group, No. (%)**				**0.026**
<65 years old	31 (47.0)	8 (23.5)	1	
≥65 years old	35 (53.0)	26 (76.5)	2.88 (1.14-7.28)	
**APACHEII Score, mean (SD)**	21.8 (7.8)	27.8 (9.7)	1.09 (1.03-1.14)	**0.002**
**VIS, No. (%)**				**<0.001**
≤120	42 (63.6)	6 (17.6)	1	
>120	24 (36.4)	28 (82.4)	8.17 (2.96-22.52)	
**CRRT, No. (%)**				**<0.001**
No	37 (56.1)	4 (11.8)	1	
Yes	29 (43.9)	30 (88.2)	9.57 (3.03-30.25)	
**Day-1 volume, No. (%)**				**<0.001**
0-1000	32 (48.5)	6 (17.6)	1	
1000-2000	21 (31.8)	5 (14.7)	1.27 (0.34-4.70)	
2000-3000	9 (13.6)	8 (23.5)	4.74 (1.30-17.24)	
>3000	4 (6.1)	15 (44.1)	20.00 (4.90-81.59)	
**Platelet, No. (%)**				**0.024**
≥100	39 (59.1)	11 (32.4)	1	
50-100	13 (19.7)	8 (23.5)	2.18 (0.72-6.59)	
<50	14 (21.2)	15 (44.1)	3.80 (1.41-10.21)	
**Lactate, median (IQR), mmol/L**	3.9 (2.0-6.7)	7.1 (2.9-15.7)	1.16 (1.06-1.26)	**0.007**

Table S4 Covariates for the baseline risk prediction model for day-14 mortality

Variable	Survivors (n=53)	Non-survivors (n=47)	Odds Ratio (95% CI)	*P*-value
**Age Group, No. (%)**				**0.003**
<65 years old	28 (52.8)	11 (23.4)	1	
≥65 years old	25 (47.2)	36 (76.6)	2.88 (1.14-7.28)	
**APACHEII Score, mean (SD)**	21.8 (7.8)	27.8 (9.7)	1.09 (1.03-1.14)	**0.002**
**VIS, No. (%)**				**<0.001**
≤120	35 (66.0)	13 (27.7)	1	
>120	18 (34.0)	34 (72.3)	8.17 (2.96-22.52)	
**CRRT, No. (%)**				**<0.001**
No	37 (56.1)	4 (11.8)	1	
Yes	29 (43.9)	30 (88.2)	9.57 (3.03-30.25)	
**Day-1 volume, No. (%)**				**<0.001**
0-1000	32 (48.5)	6 (17.6)	1	
1000-2000	21 (31.8)	5 (14.7)	1.27 (0.34-4.70)	
2000-3000	9 (13.6)	8 (23.5)	4.74 (1.30-17.24)	
>3000	4 (6.1)	15 (44.1)	20.00 (4.90-81.59)	
**Platelet, No. (%)**				**0.040**
≥100	32 (60.4)	18 (38.3)	1	
50-100	11 (20.8)	10 (21.3)	2.18 (0.72-6.59)	
<50	10 (18.9)	19 (40.4)	3.80 (1.41-10.21)	
**Lactate, median (IQR), mmol/L**	3.9 (2.0-6.7)	7.1 (2.9-15.7)	1.16 (1.06-1.26)	**0.007**
**Total bilirubin**				**0.020**
≤34	38 (71.7)	23 (48.9)	1	
>34	15 (28.3)	24 (51.1)	2.64 (1.16-6.05)	
**Fibrinogen**				**0.045**
<2.0	8 (15.1)	16 (34.0)	1	
2.0-4.0	20 (37.7)	18 (38.3)	0.45 (0.16-1.30)	
>4.0	25 (47.2)	13 (27.7)	0.26 (0.09-0.77)	

Table S5 Covariates for the baseline risk prediction model for day-28 mortality

Variable	Survivors (n=43)	Non-survivors (n=57)	Odds Ratio (95% CI)	*P*-value
**Age Group, No. (%)**				**0.030**
<65 years old	22 (51.2)	17 (29.8)	1	
≥65 years old	21 (48.8)	40 (70.2)	2.47 (1.14-7.28)	
**APACHEII Score, mean (SD)**	20.1 (7.7)	26.7 (8.8)	1.10 (1.04-1.16)	**<0.001**
**VIS, No. (%)**				**<0.001**
≤120	30 (69.8)	18 (31.6)	1	
>120	13 (30.2)	39 (68.4)	8.17 (2.96-22.52)	
**CRRT, No. (%)**				**<0.001**
No	29 (67.4)	12 (21.1)	1	
Yes	14 (32.6)	45 (78.9)	5.00 (2.12-11.79)	
**Day-1 volume, No. (%)**				**0.021**
0-1000	23 (53.5)	15 (26.3)	1	
1000-2000	11 (25.6)	15 (26.3)	2.09 (0.76-5.76)	
2000-3000	5 (11.6)	12 (21.1)	3.68 (1.08-12.58)	
>3000	4 (9.3)	15 (26.3)	5.75 (1.60-20.69)	
**Lactate, median (IQR), mmol/L**	3.7 (2.0-5.8)	5.3 (2.9-12.0)	1.15 (1.04-1.27)	**0.01**

Figure S1. Mortality by different classification of ACP among septic shock patient within first 24 hours.

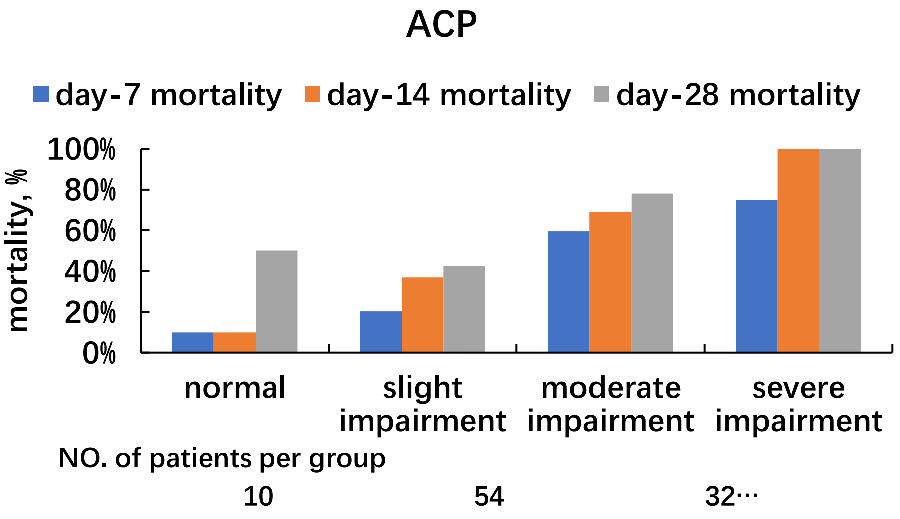

Figure S2 Covariates for Cox proportional hazards regression analyses of day-28 mortality (ACP)

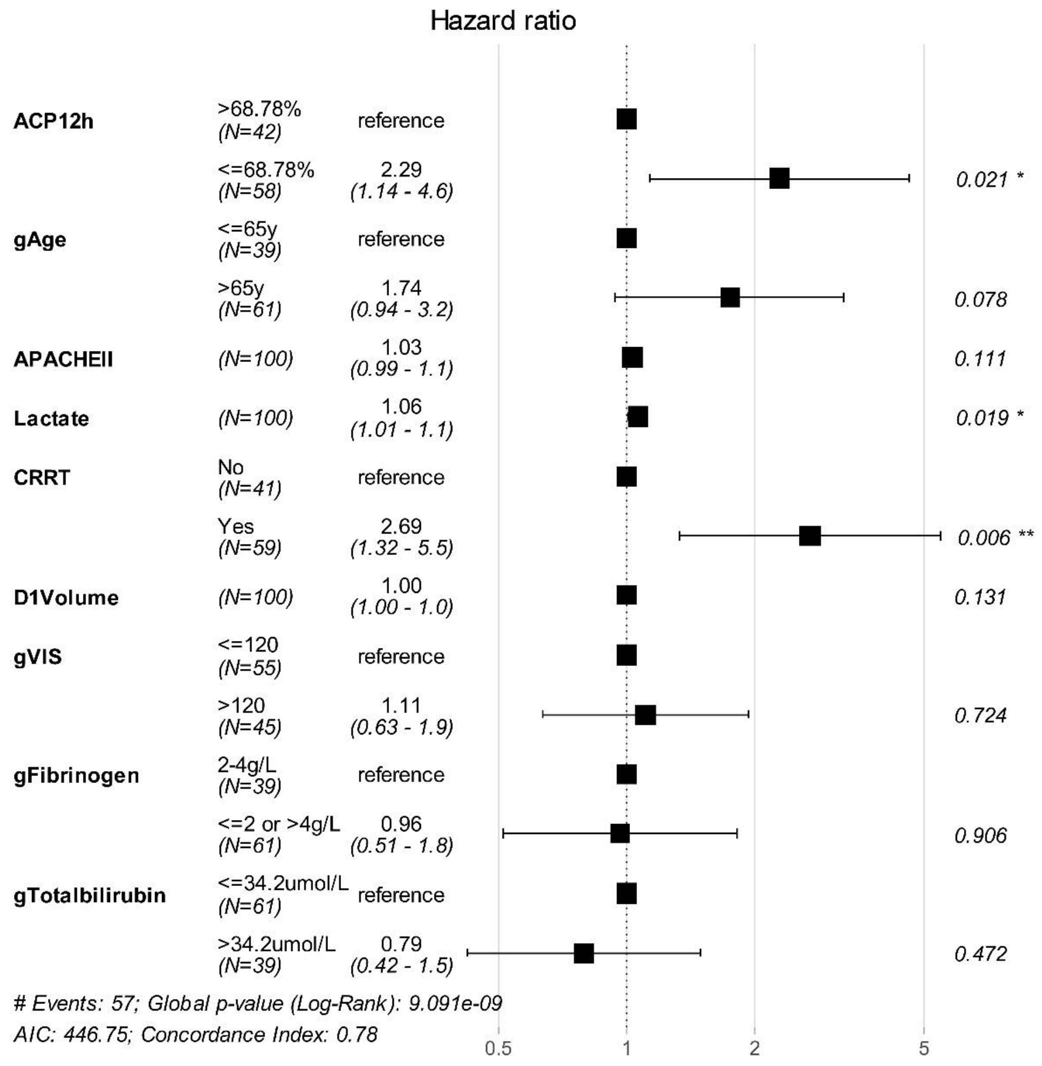

Figure S3 Covariates for Cox proportional hazards regression analyses of day-28 mortality (CI)

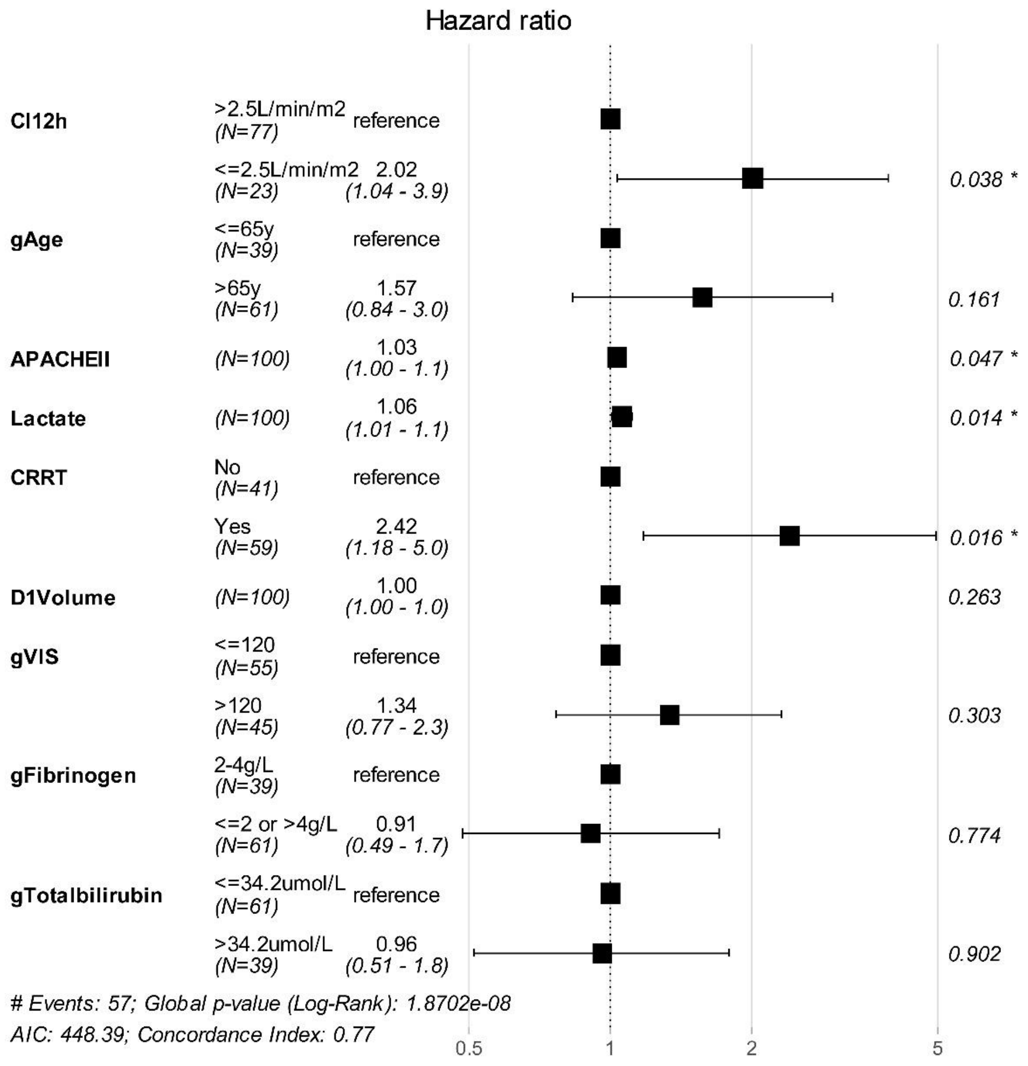

Figure S4 Covariates for Cox proportional hazards regression analyses of day-28 mortality (CPI)

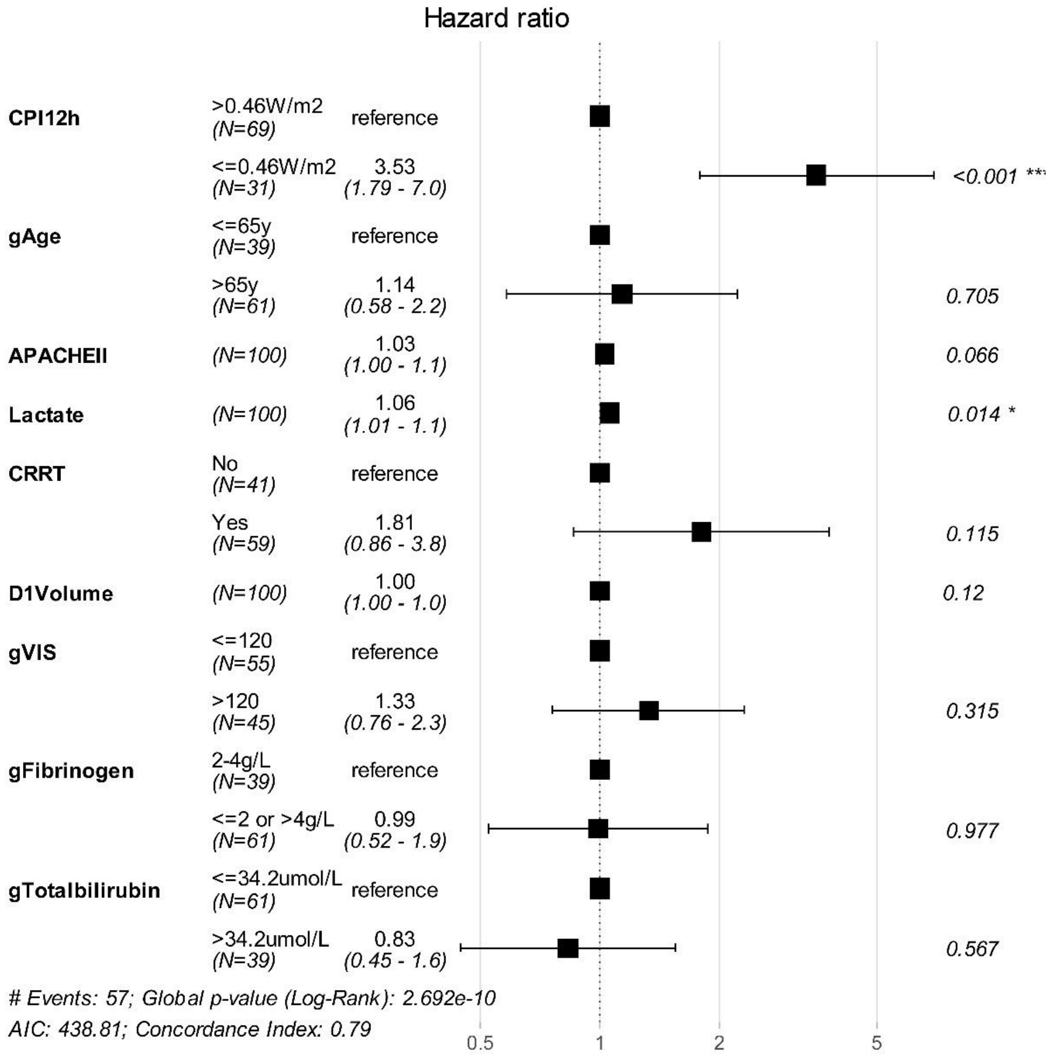

Figure S5 Cox proportional hazards regression analyses of day-28 mortality according to classification of ACP in patients with septic shock.

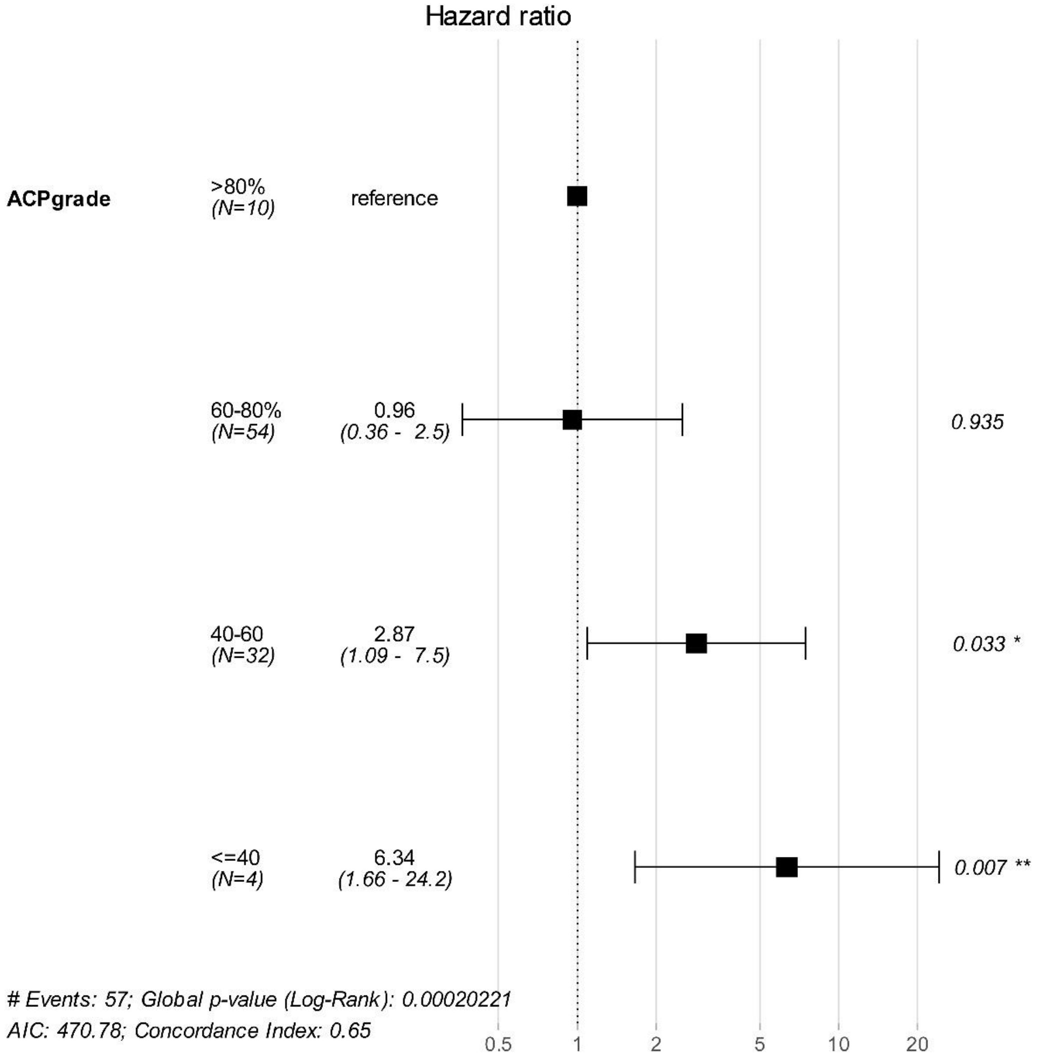

Figure S6 Cox proportional hazards regression analyses of day-28 mortality according to classification of parameters in patients with septic shock.

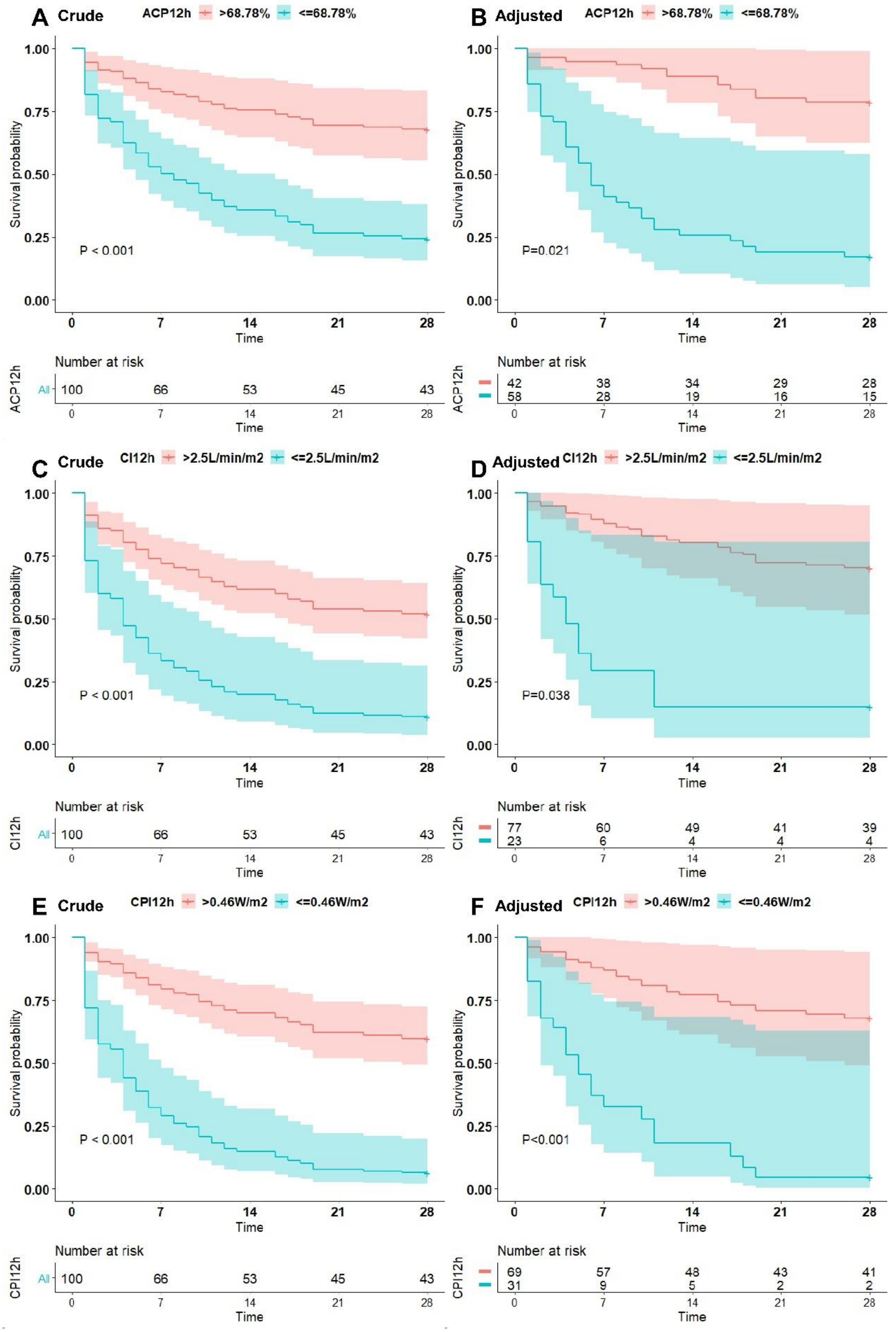

Figure S7 proportional hypothesis test for Cox proportional hazards regression model (ACP)

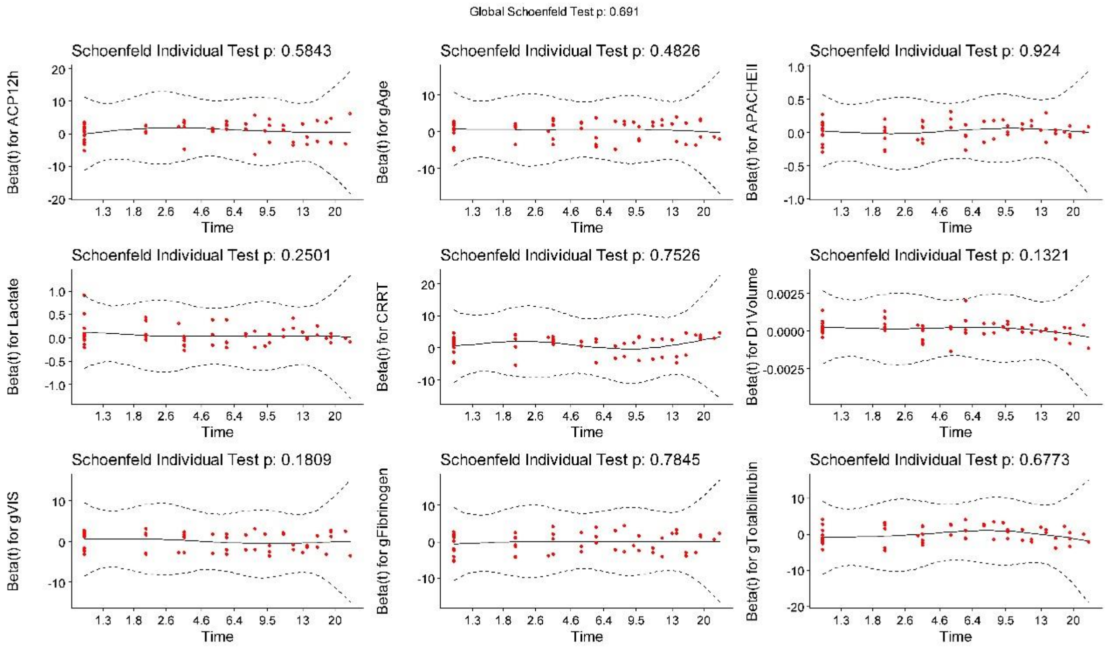

Figure S8 proportional hypothesis test for Cox proportional hazards regression model (CI)

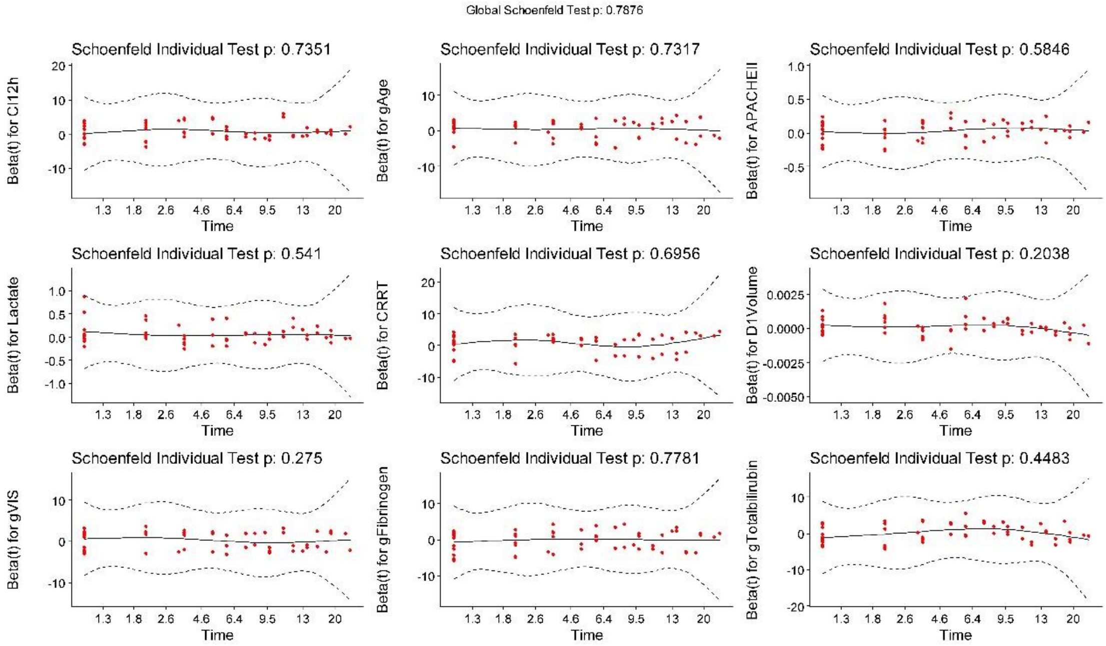

Figure S9 proportional hypothesis test for Cox proportional hazards regression model (CPI)

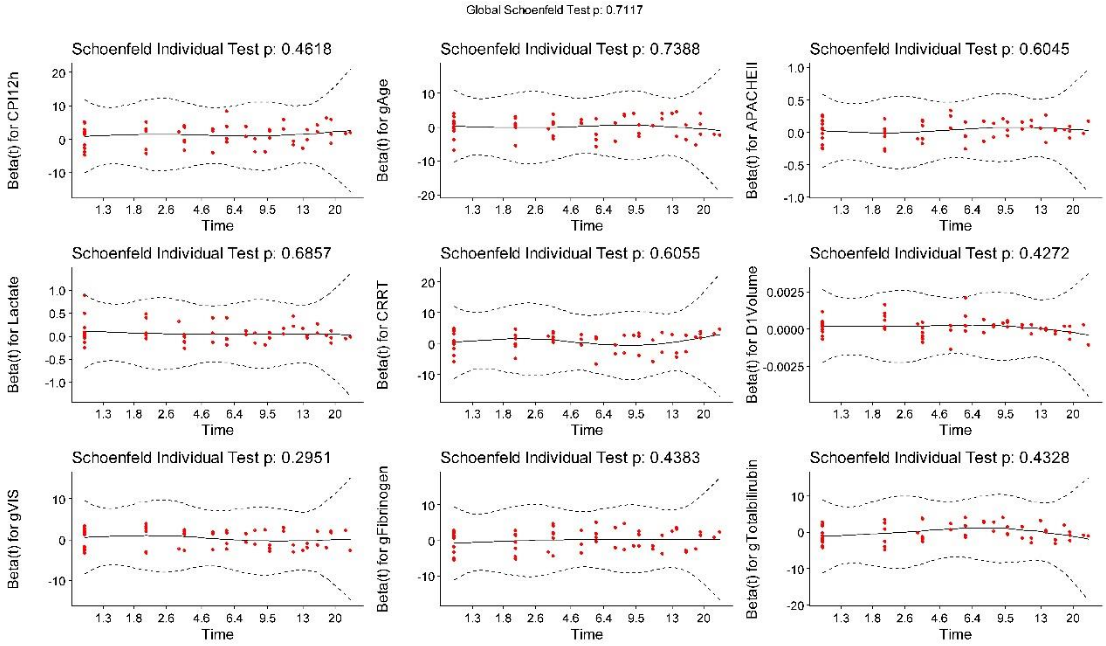

Supplement: Supplementary file 1 — Additional file 1. Supplement file. [file 40560_2021_549_MOESM1_ESM.pdf]
